# A Dynamic Bayesian Network model for the simulation of Amyotrophic Lateral Sclerosis progression

**DOI:** 10.1186/s12859-019-2692-x

**Published:** 2019-04-18

**Authors:** Alessandro Zandonà, Rosario Vasta, Adriano Chiò, Barbara Di Camillo

**Affiliations:** 1 Department of Information Engineering, University of Padova, Gradenigo 6/b, 35131 Padova, Italy; 2 Department of Neuroscience, University of Torino, 10124 Torino, Italy

**Keywords:** Dynamic Bayesian network, Amyotrophic lateral sclerosis, Simulation, Prediction, Survival, MITOS, Stratification

## Abstract

**Background:**

Amyotrophic lateral sclerosis (ALS) is an adult-onset neurodegenerative disease progressively affecting upper and lower motor neurons in the brain and spinal cord. Mean life expectancy is three to five years, with paralysis of muscles, respiratory failure and loss of vital functions being the common causes of death. Clinical manifestations of ALS are heterogeneous due to the mix of anatomic regions involvement and the variability in disease course; consequently, diagnosis and prognosis at the level of individual patient is really challenging. Prediction of ALS progression and stratification of patients into meaningful subgroups have been long-standing interests to clinical practice, research and drug development.

**Methods:**

We developed a Dynamic Bayesian Network (DBN) model on more than 4500 ALS patients included in the Pooled Resource Open-Access ALS Clinical Trials Database (PRO-ACT), in order to detect probabilistic relationships among clinical variables and identify risk factors related to survival and loss of vital functions. Furthermore, the DBN was used to simulate the temporal evolution of an ALS cohort predicting survival and the time to impairment of vital functions (communication, swallowing, gait and respiration). A first attempt to stratify patients by risk factors and simulate the progression of ALS subgroups was also implemented.

**Results:**

The DBN model provided the prediction of ALS most probable trajectories over time in terms of important clinical outcomes, including survival and loss of autonomy in functional domains. Furthermore, it allowed the identification of biomarkers related to patients’ clinical status as well as vital functions, and unrevealed their probabilistic relationships. For instance, DBN found that bicarbonate and calcium levels influence survival time; moreover, the model evidenced dependencies over time among phosphorus level, movement impairment and creatinine. Finally, our model provided a tool to stratify patients into subgroups of different prognosis studying the effect of specific variables, or combinations of them, on either survival time or time to loss of autonomy in specific functional domains.

**Conclusions:**

The analysis of the risk factors and the simulation allowed by our DBN model might enable better support for ALS prognosis as well as a deeper insight into disease manifestations, in a context of a personalized medicine approach.

**Electronic supplementary material:**

The online version of this article (10.1186/s12859-019-2692-x) contains supplementary material, which is available to authorized users.

## Background

Amyotrophic lateral sclerosis (ALS) is an adult-onset neurodegenerative disease characterised by the progressive involvement of motor neurons [1–6]. The incidence of ALS in Europe is 2–3 cases per 100,000 person-years [2], while in the United States is approximately 1–2/100,000 with a prevalence of 4–6/100,000 [6]; in east and south Asia the incidence is lower (~ 0.8 and ~ 0.7 cases per 100,000 person-years, respectively) [2]. The clinical hallmark of ALS is the degeneration of both upper motor neurons (originating in the motor cortex and descending to the brainstem and the spinal cord) and lower motor neurons (connecting the brainstem and the spinal cord to the muscle). Several studies suggested that many molecular and cellular mechanisms might be implicated in a cell damage cascade leading to motor neuron death, including altered calcium homeostasis, glutamate excitotoxicity, aggregation of ubiquitylated proteinaceous inclusions in motor neurons, prion-like spreading, and RNA metabolism defects and toxicity [2, 4, 6]. Accumulating evidences suggest that the interplay between genetic and environmental factors might have an important role in ALS causation [1–5], although the pathophysiological processes underlying ALS is still unclear.

Commonly, the primary symptoms of ALS are associated with motor deficits, such as foot drop, spasticity and difficulty walking and lifting arms due to weakness, followed by dysphagia, impaired fine movements and emotional lability [2]. The 70% of ALS patients exhibit limb-onset disease and the 30% of cases present bulbar-onset disease; mean life expectancy after symptom onset is three to five years, with respiratory failure being the most common cause of death [1, 7]. The variability of onset site, the relative mix of upper and lower motor neuron involvement, the rate and the pattern of progression result in heterogeneous ALS phenotypes [8] and a challenging diagnosis. Moreover, since no diagnostic test for ALS is available, a two-step procedure is commonly adopted in clinical practice to diagnose ALS: the exclusion of other conditions that present similar features to ALS and the definition of “diagnostic certainty” based on specific criteria [2]. In detail, El Escorial and Airlie House are the criteria with the wider agreement among clinicians. The diagnosis according to these criteria is based on the identification of the extent and spreading of upper and lower motor neuron signs, supported by neurophysiological and imaging data. However, a timely diagnosis is challenging since these criteria require the history of disease progression.

ALS is a disease with a wide heterogeneity in terms of clinical manifestations, rate and pattern of progression, and ultimately survival; therefore, the identification of predictive biomarkers is needed for stratification and prognosis support. Several systems for monitoring ALS staging have been designed, including the ALS Functional Rating Scale Revised (ALSFRS-R), the King’s clinical staging system [9] and the ALS Milano-Torino functional staging system (MITOS) [10]. The ALSFRS-R is based on 12 items rated on a 0–4 point scale evaluating bulbar functions, fine and gross motor skills and respiratory functions; MITOS encodes the loss of autonomy in four key domains that are included in ALSFRS-R: bulbar functions (communication and swallowing), fine and gross motor skills (such as walking) and respiratory functions. King’s maps ALSFRS-R domains to equivalent body regions affected by ALS and encodes the occurrence of nutritional or respiratory dysfunction.

Most of the costs associated with ALS is related to the management of the disease complications. In the clinical context of the patient management and treatment, it is thus of particular interest to predict the dynamics of ALS and to perform simulation analyses about the potential effectiveness of specific therapeutic interventions.

In this work, we focus our attention on the problem of deriving a probabilistic simulator of the progression of ALS and its complications, by learning a Dynamic Bayesian Network (DBN) model from a large public dataset such as the *Pooled Resource Open-Access ALS Clinical Trials Database* (PRO-ACT). The model can be used to predict the progression of single patient or of a population of patients. A major strength of our approach is the explicit representation of the relations between the different risk factors and the pathways along which each risk factor influences the clinical outcomes. This could be used to tailor better patient-specific interventions, by studying the temporal evolution of risk factors and the estimated alteration of the different variables’ pathways.

## Methods

### The PRO-ACT database

The *Pooled Resource Open-Access ALS Clinical Trials Database* (PRO-ACT) is an open-access database retrievable at https://nctu.partners.org/ProACT, which includes records of more than 10,700 ALS patients from different clinical trials, providing over 2,869,973 longitudinally collected data measurements. The PRO-ACT includes a broad spectrum of information assessed over subsequent screening visits such as demographics, family history, forced and slow vital capacity, laboratory data (e.g., basophil, blood and platelets count), concomitant medication and Riluzole use, ALSFRS-R and vital signs (e.g., pulse, blood pressure). PRO-ACT was funded by ALS Therapy Alliance and was developed in the context of DREAM Phil Bowen ALS prediction Prize4Life in 2012 [11]. During the following years, Prize4Life included more than 9000 new ALS patients into PRO-ACT. In 2015, the DREAM ALS Stratification Prize4Life Challenge utilized this growth in data to develop tools for the identification of subgroups of ALS patients with distinct clinical outcomes. In December 2015, new clinical trials were added into the PRO-ACT database that accounts for 10,723 subjects for a total of 2,869,973 records. The dataset includes static variables, which are either time-independent covariates (e.g.*,* gender) or data collected at first visit only (e.g.*,* age at onset), and dynamic variables that are time-dependent measurements collected over subsequent visits. The latest version of PRO-ACT database (April 1st, 2016) was used in this work.

### Preprocessing

We excluded variables that were missing for more than 50% of the subjects. Measurement units were then homogenized. Finally, we filtered out patients for which time of onset or multiple visits were not available. We then split the data into a training set for developing the Dynamic Bayesian Networks, and a validation set (around 25% of subjects) for validating our model, for a total of 3970 and 987 subjects in the training and in the validation set, respectively (see Additional file 1 for data details). The split between training and test was performed stratifying patients for number of deaths, which resulted in a well-stratified split also for other variables (see Additional file 1). ALSFRS-R was converted into MITOS using the algorithm proposed in [10]: the MITOS scale encodes the loss of independence in the four key domains (movement, communication, swallowing and breathing) on the ALSFRS-R. Values equal to zero are assigned to the domains that are not impaired, whereas values equal to one are assigned to the domains in which patient’s independence is lost. Since the impairment of a domain is not reversible, MITOS was then set to monotonically increase over time, with the purpose of fixing some mistakes/variability occurred during the ALSFRS-R clinical compilation.

Data were quantized using the clinical thresholds as given in PRO-ACT website (https://nctu.partners.org/ProACT/) and as reported in Additional file 1. Only age at onset, time between onset and the start of the trial, and time between visits were quantized using tertiles so to guarantee a number of intervals similar to the majority of the other variables and equal distributions of examples in the bins. For instance, age at onset was discretized in three levels based on its distribution: lower than 51, between 51 and 61, or higher than 61.

The preprocessing was implemented by in-house R scripts.

### Dynamic Bayesian networks

A Bayesian Network [12, 13] is a mathematical representation of a joint probability distribution of a set of random variables based on a set of conditional independence assumptions. The structure of a Bayesian Network is a directed acyclic graph (DAG) such that each random variable corresponds to a node and the influence of one node (parent) on another (child) corresponds to a directed edge. The network structure induces a set of conditional probability distributions (CPDs), since each variable is a probabilistic function of its parents. The network structure annotated with its CPDs, completely defines a Bayesian Network (BN). The extension of a BN to model dynamic processes is a Dynamic Bayesian Network (DBN), which describes the dependencies among the variables over time [13]. Nodes in a DBN are still connected through a DAG; however, DBNs allow encoding cycles and feedbacks between variables when considering their relationships over different time slices. The key assumption is that the probability distributions describing the temporal dependencies are time invariant and DBNs relate variables to each other over a discrete number of time steps, called time slices. For example, weight at time (*t – 1*) influences the state of weight at time (*t*).

To learn a DBN model from the data we used *bnstruct* [14], an R package that performs structure and parameter learning on discrete/categorical data even in the presence of missing values, which is the case of our data and a common situation in the clinical context. *Bnstruct* makes use of state-of-the-art algorithms for network learning and provides also methods for bootstrap re-sampling of the data and inference.

Constraints can be applied to the network structure learned by *bnstruct* to codify the domain knowledge. To this purpose, variables were divided into six layers (Table 1) and edges from lower to higher layers were forbidden to exclude any clinically or biologically non-sense relations among variables, such as the dependence of gender from the site of onset. It is also possible to constrain the influence of upper layers to lower layers. In our case, variables at time *t* were allowed to influence each other, and variables at time *t-*1 were allowed to affect both survival and variables at time *t*. Variables in layer 1 (gender, age at onset) were allowed to affect only variables in layer 2 (onset site and time between onset and diagnosis), variables in layer 3 (Riluzole, placebo/treatment intake), variables at time *t* and survival. Variables in layer 2 were allowed to influence only variables in layer 3, variables at time *t* and survival.

**Table 1:** Layering. Layering and type of variables in the DBN

Layer	Variable	Type
1	Gender, age at ALS onset	Static
2	Onset site, onset delta (start of the trial - onset)	Static
3	Riluzole intake, placebo/treatment	Static
4	Variables at time *t-1*	Dynamic
5	Variables at time *t*, TSO	Dynamic
6	Survival	Static

The dynamic variables of the PRO-ACT dataset were collected on a non-uniform, but quite inhomogeneous, time grid (training set: 44 ± 20 days; validation set: 44 ± 21 days), because different patients were visited at different time intervals, with their specific frequency of visits. The time window during which each patient was observed in the trial is also non-uniform (training set: 549 ± 244 days; validation set: 549 ± 244 days). To account for different observation-windows and different time-grids among subjects, we added, as additional variable in the network, the cumulative time since onset (TSO), with the possibility of influencing variables at time *t* and survival.

The DBN was inferred on the training data through a two-step iterative procedure: i) learning the graph topology (i.e.*,* dependencies among nodes) and ii) learning the parameters of each CPD (i.e.*,* distribution over the values of a node given each possible joint assignment of values to its parents), computed as Maximum-a-Posteriori estimates.

The DBN structure learning was performed using the Maximum Minimum Hill-Climbing (MMHC) algorithm [15, 16]. MMHC is an hybrid algorithm combining techniques from both constraint-based and search-and-score approaches for learning Bayesian networks from data. The algorithm is based on two steps: first, the Maximum Minimum Parents Children (MMPC) [17] algorithm infers the skeleton of the network, and then Hill Climbing [16] algorithm (HC) reconstructs edges orientation. MMPC uses a constraint-based technique that tests the conditional independence and measures the strength of relationship between pairs of variables. MMPC identifies an edge between the nodes X and Y if and only if they are not independent given any subset of nodes S; at the end, MMPC provides the parents and children set of each node. Subsequently, MMHC performs Hill-Climbing search in the space of Bayesian networks starting from the initial configuration provided by MMPC. A score function quantifying how well the network fits the data is computed at this first step of HC. The score metric that was adopted in the present study was the Bayesian Information Criterion (BIC), which trades off both the likelihood and the model complexity. The BIC score is defined as:

$$ {score}_{BIC}\left(G:D\right)=\ell \left({\vartheta}_G:D\right)-\frac{logM}{2} Dim\left[G\right] $$

Where *ℓ* is the likelihood function of the network G, *ϑ* are the parameters maximizing the likelihood, *M* is number of observations, and *Dim*[*G*] is the number of independent parameters in G. In detail:

$$ Dim(G)=\sum \limits_{i=1}^N\left({R}_i-1\right){Q}_i $$

Where N is the number of nodes in G, Q_i_ is the number of possible combinations of values for the parents of node *i* and R_i_ is number of possible values for variable *i*.

Over subsequent steps of Hill-Climbing algorithm, all the neighbours in the G space are considered and the BIC score is computed for each of them, after adding, deleting or reversing the direction of the edges recursively. At each step, the change in the network G that results in the largest increase of the score is then applied. The main difference between MMHC and the standard search-and-score techniques is that the search is constrained to only add an edge if it was identified by MMPC in the first stage. The algorithm stops when either the score does not improve with any change in the network or a specific number of iterations (15 in our case) has been reached. Thus, the structure learning phase provided the topology of the DBN with the highest probability to have generated the data. Subsequently, a Maximum a Posteriori estimation computed the set of parameters of the conditional probability distribution at each node.

We adopted MMHC algorithm for DBN learning because of the higher BIC score with respect to the standard HC.

### ALS simulation

In a DBN, the temporal evolution of the analysed process can be reconstructed by knowing the temporal dependencies represented in the DBN graph [13, 18] and the data at time 0. The DBN learnt from the training data was thus used to simulate the ALS temporal evolution in terms of MITOS changes over time and survival. The conditional probability distributions inferred on the training set encode variable dependencies over time, thus the temporal evolution of each patient was simulated by sampling, at each discrete time point, the state of the patient conditioned on his/her state in the previous time point in accordance to the CPDs. In detail, we ran a simulation starting from the first visit of each of the 987 patients in the validation set and we let the system evolve for 30 time slices or until an in-silico patient death occurred. For each patient, 400 different simulations were run for a total of 394,800 in-silico patients, so to have a probabilistic distribution of clinical variables evolution over time.

### Model assessment

The simulation performance was measured for different time points (month 12, 24, 36 and 42) as the difference between the percentage of deceased patients in simulated and real data (Table 2). The same was done for time-of-events related to the functional impairment in the MITOS domains.

**Table 2:** Simulation error. Simulation error (%) computed at different time points as the difference between the percentage of real and simulated patients experiencing either the impairment in the four MITOS domains or death

Months	12	24	36	42
Outcomes				
MITOS Movement	2.26	16.71	4.27	0.63
MITOS Communicating	0.74	1.86	0.97	0.72
MITOS Swallowing	0.12	1.20	5.37	1.66
MITOS Breathing	0.09	2.85	1.85	7.61
Survival time	2.53	7.15	1.84	2.76

In addition, the ability of DBN model to rank subjects based on their risk (of death and of functional impairment based on the four MITOS domains) was assessed in terms of Areas Under the ROC (AU-ROC) Curve calculated based on events observed at different time points (months 12, 24, 36 and 42). The greater the AU-ROC (range 0–1), the more accurate is our simulation.

To further validate the DBN approach, we adopted a cross-validation (CV) schema, in order to detect possible bias effects (i.e.*,* overfitting, selection bias) and warrant adequate accuracy of the DBNs on novel data. In detail, we considered the entire dataset (*tot_ds* of 4957 subjects) and implemented a 10-fold cross-validation approach as follows, for each of the 10 iterations:

1. First, *tot_ds* was split in 9/10 as training set (*CV_tr*) and 1/10 as validation set (*CV_val*)
2. *CV_tr* was used for DBN development
3. The DBN model, learnt at step 2, was used to simulate patients evolution as explained in paragraph “ALS simulation” and compared with real patients progression as observed in *CV_val* in terms of Areas Under the ROC Curve calculated based on events observed at different time points (months 12, 24, 36 and 42).

We finally computed the mean and the standard deviation of the 10 AU-ROCs obtained for each time point.

## Results

**Fig. 1:**
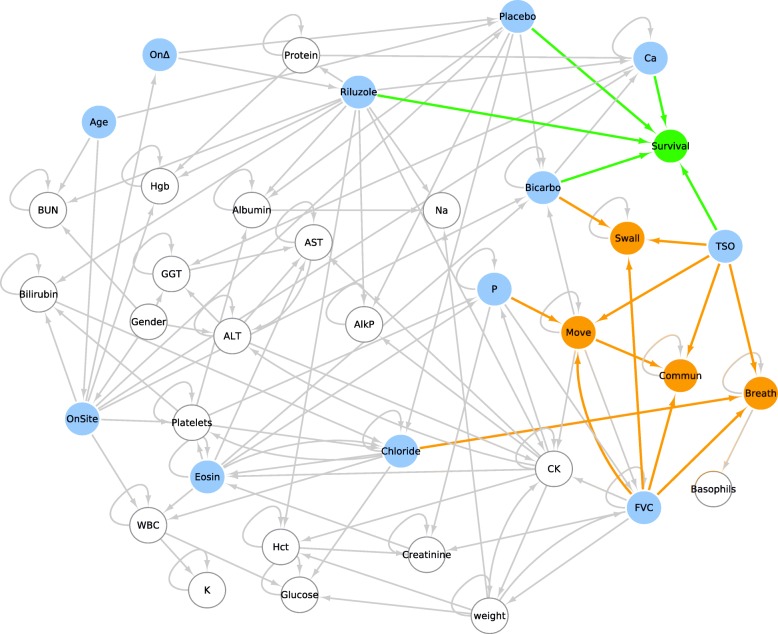
Subset of the DAG obtained on training dataset*.* Only nodes with at least one direct edge are shown. MITOS items and survival are reported in orange and green, respectively; variables with direct or indirect influence on either survival or MITOS items are evidenced in cyan. ALT: alanine amino transferase, AST: aspartate amino transferase, AlkP: alkaline phosphatase, BUN: blood urea nitrogen, OnΔ: onset delta (time between onset and the first time the patient was tested in a trial), OnSite: onset site, P: phosphorus, RBC: red blood cells, AST: aspartate amino transferase, Ca: Calcium, CK: creatine kinase, Eosin: Eosinophils, FVC: forced vital capacity, GGT: gamma-glutamyltransferase, K: Potassium, Hct: hematocrit, Hgb: hemoglobin, TSO: time since onset, WBC: white blood cells. (PDF 430,5 KB)

The DBN learnt on the training dataset encoded the probabilistic relationships among variables as a DAG of 68 nodes and 127 edges (Fig. 1). Some of the identified dependencies were expected, such as the dependence of disease severity stage on Forced Vital Capacity (FVC), which is a common clinical measure for ALS progression [19]. The DBN clearly evidenced that the loss of independence on the four MITOS domains was related to the changes in FVC along time. The DBN model also confirmed the relationship between gender and site of onset; indeed, most women have bulbar-onset disease and men have higher propensity for spinal-onset disease [2, 8]. A relationship between the respiratory impairment and chloride levels was also found, which is an expected result since chloride has been related with respiratory symptoms that are predictive of death [20]; in addition, chloride represents the degree of respiratory acidosis [21] that is consequence of respiratory failure.

Notably, our analysis revealed also new dependencies among variables. For instance, our model showed a probabilistic dependence of survival time on the levels of calcium, which might be based on an ALS-related dysfunction in the endoplasmic reticulum (ER) of motor neurons that is a major source of calcium [22]. Moreover, the DBN modelled the dependencies over time among phosphorus levels, movement impairment and creatinine, which has been already indicated as a marker of ALS outcome [23]. In detail, it has been shown that creatinine at diagnosis is a reliable prognostic factor of motor dysfunction in ALS, but its evolution along ALS progression is still unclear. Both creatinine and phosphorus were identified as non-standard predictive features in recent studies [11, 24], which claimed the need to further explore their potential predictive properties. Our analysis showed that phosphorus mediated the role of creatinine on disease progression and modelled how the changes in the levels of these two hematological factors can affect each other as well as movement ability. In addition, the DBN identified a probabilistic relationship between the survival time and bicarbonate, which has been recently detected as a possible biomarker to predict the death risk [25]. Furthermore, our model evidenced the impact of experimental medication or Riluzole intake on both survival and ALS progression.

**Fig. 2:**
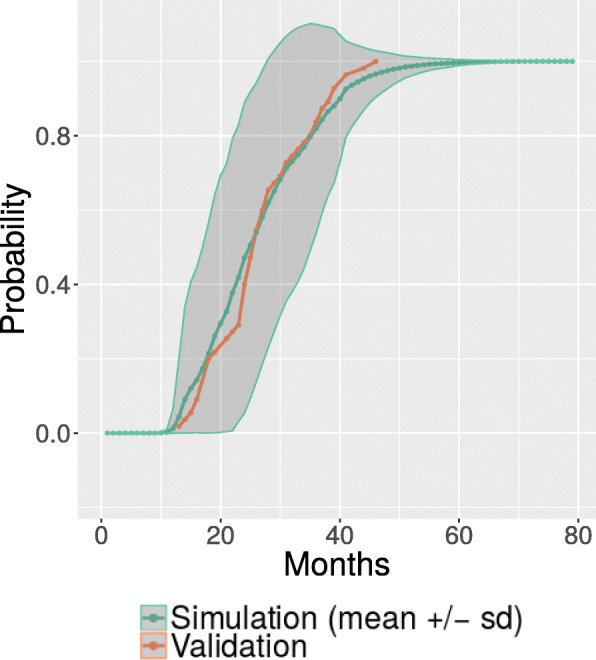
Simulated probability of death over time. Probability of death over time in the validation dataset (orange line) and in the simulated population (green line: mean values over population; shaded region: standard deviation), based on probabilities modelled by DBN. (PDF 29,3 KB)

As explained in paragraphs “ALS simulation” and “Model assessment”, the true dynamics of the patients in the validation set were compared with the ones predicted by simulation. Figure 2 shows the survival time of the simulated vs. the true validation data population; the comparison between the probability of death in real (validation set) and simulated ALS population is reported, showing that the DBN model provides a precise simulation of survival.

**Fig. 3:**
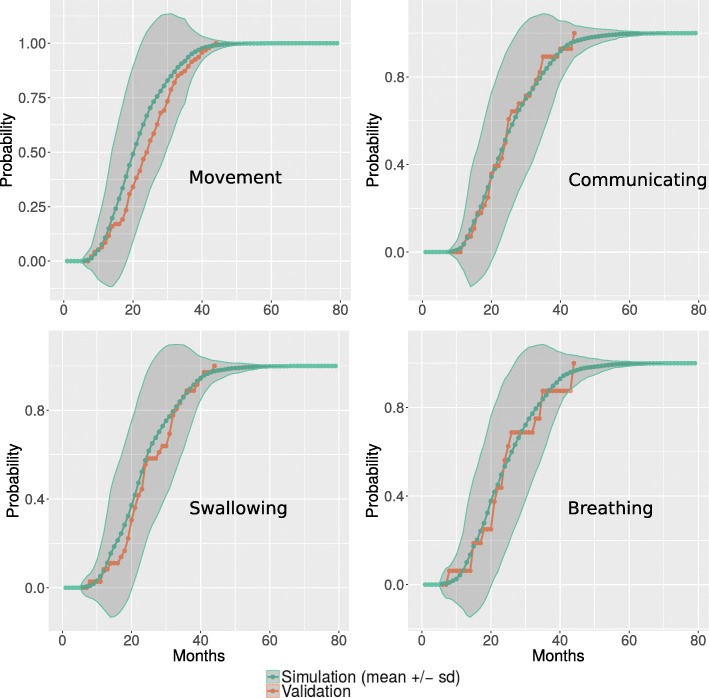
Simulated probability of MITOS impairment over time. Distribution of the temporal evolution of MITOS items in the validation set (orange line) and in the prediction (green line: mean values over population; shaded region: standard deviation), based on probabilities modelled by DBN*.* (PDF 66,4 KB)

A good correspondence between simulated and real distribution was also found for all the four MITOS domains over the first 80 months of ALS progression. Figure 3 reports the probability distribution of impairment in the four functional domains encoded by MITOS, in the true vs. the simulated ALS populations.

Prediction error (in %) and AU-ROC at different time points (paragraph “Model Assessment”) were also computed, in order to assess the simulation performance over all the simulated time points. A good correspondence between real and simulated ALS progression was evidenced for all the four MITOS domains as well as for survival time (Tables 2 and 3). The DBN model was thus able to efficiently predict the times to the impairment of the functional domains coded by MITOS (breathing/swallowing/movement/communicating ability), as well as the survival time.

**Table 3:** AU-ROCs. AU-ROCs computed for each simulated time to MITOS impairment and predicted survival time

AU-ROC				
	Months			
Outcomes	12	24	36	42
MITOS Movement	0.92	0.77	0.91	0.93
MITOS Communicating	0.91	0.95	0.90	0.98
MITOS Swallowing	0.89	0.88	0.89	0.95
MITOS Breathing	0.90	0.85	0.96	1.00
Survival time	0.96	0.9	0.94	0.92

Moreover, we adopted a 10-fold Cross-Validation (CV) schema in order to control the risk of overfitting and bias effects of our approach. Table 4 reports the average AU-ROC of the 10 different DBN models that were obtained inside the CV. The simulation performance of the DBN learnt on the training set was comparable with the mean performance of the models from the CV, except for the performance on MITOS breathing of the first 24 months. In this scenario, the models from CV achieved fairly different AU-ROCs (note the high standard deviation), because the split between training and validation inside the CV was performed not stratifying patients for number of breathing impairment events. Furthermore, these events are commonly experienced at the last stages of the disease, thus the DBN could learn only on a few examples over the first 24 months and the performance was lower than for further time points.

**Table 4:** AU-ROCs for the CV models. Average AU-ROCs of the DBNs obtained from the CV computed for each simulated time to MITOS impairment and predicted survival time

AU-ROC mean ± standard deviation				
	Months			
Outcomes	12	24	36	42
MITOS Movement	0.91 **±** 0.09	0.82 **±** 0.04	0.84 **±** 0.05	0.87 **±** 0.07
MITOS Communicating	0.89 **±** 0.09	0.71 **±** 0.14	0.77 **±** 0.07	0.81 **±** 0.08
MITOS Swallowing	0.63 **±** 0.32	0.77 **±** 0.11	0.81 **±** 0.07	0.84 **±** 0.11
MITOS Breathing	0.63 **±** 0.51	0.66 **±** 0.06	0.75 **±** 0.08	0.84 **±** 0.08
Survival time	0.87 **±** 0.18	0.77 **±** 0.11	0.85 **±** 0.04	0.85 **±** 0.07

Overall, we ensured that our model was not performing well only on this specific training-validation scenario, rather it warranted comparable accuracy on different data partitions.

**Fig. 4:**
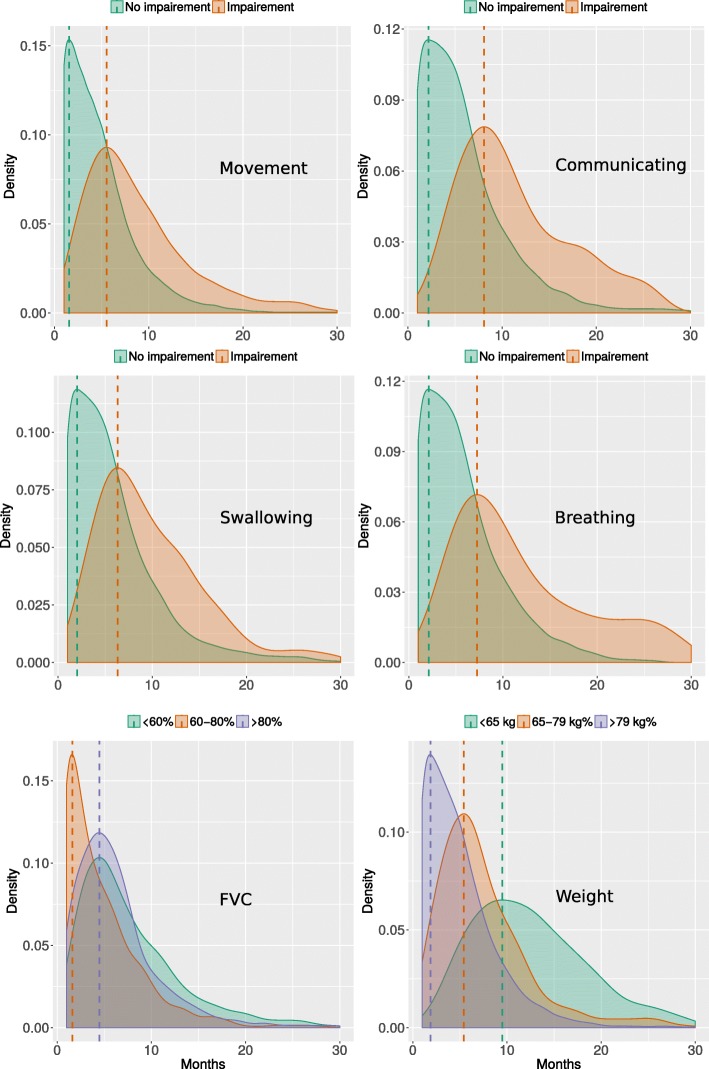
Patient-level probability of MITOS impairment over time. Density plot of the probable values (color-coded) of MITOS items, FVC and weight predicted along 30 months (x-axis) for a single patient. 400 different temporal evolutions were generated for each simulated patient so to have a distribution of variable values in time. (PDF 134,7 KB)

**Fig. 5:**
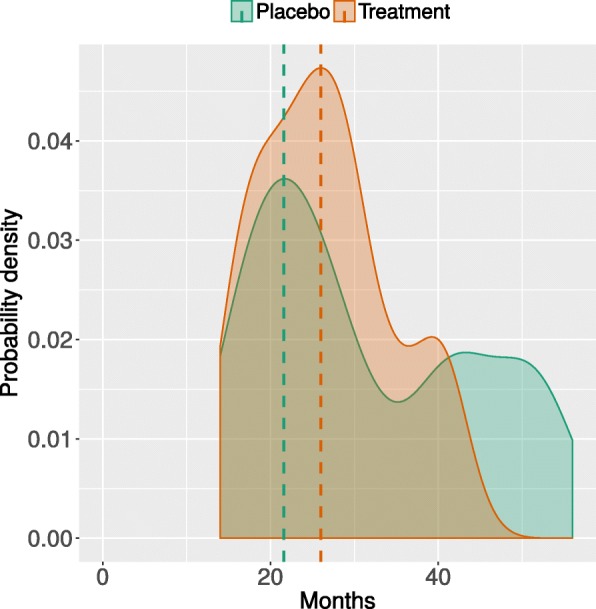
Patients’ stratification. Density plot of the probable survival time for two ALS populations with placebo intake (green curve) and experimental medication intake (orange curve). 100 different temporal evolutions were generated so to have a distribution of variable values in time. (PDF 17,7 KB)

Our model can be used also to simulate, for a single patient, the time-dependent probability of a variable being in a certain state. This can be useful to provide clinicians with a probabilistic prognostic tool, useful to allocate resources and provide indications on therapeutic interventions. Figure 4 reports the density plot of the values of MITOS items, FVC and weight predicted for a single patient on 400 simulations. The simulation of ALS progression on a population can also be used to study the effect of specific variables, or combinations of them, on survival time or time to loss of autonomy in some functional domains (i.e., MITOS changing from 0 to 1), thus providing a tool for the stratification of patients into subgroups of different prognosis. For instance, Fig. 5 reports the effect of experimental medication intake on survival time. The graph was obtained by simulating the temporal evolution of two different ALS populations treated with placebo or experimental medication, then assessing the survival time. As expected, the patients receiving placebo treatments since the onset showed shorter survival compared to the patients that were provided with experimental treatments. MITOS items distribution for the two treated populations are reported in Additional file 1.

## Discussion

This study introduces a Dynamic Bayesian Network (DBN) model for the analysis of clinical measures in Amyotrophic Lateral Sclerosis (ALS) and its application on more than 4900 ALS patients included in the Pooled Resource Open-Access ALS Clinical Trials Database (PRO-ACT). The aim of this model is to unravel the probabilistic dependencies among clinical variables over time and predict the ALS temporal evolution. Moreover, our model can be useful to stratify ALS patients into subgroups of different prognosis and to predict their most probable trajectories over time, in terms of survival and loss of autonomy in functional domains.

One of the main limitations of our work is that our DBN model requires variables discretized in a limited number of states, thus the predictions of variables progression indicate the most probable range for a variable, rather than its continuous value.

Nevertheless, as far as we know our model is the first DBN used to simulate ALS progression in a probabilistic, dynamic setting. Differently from other predictive methods, which allow predicting survival time or, more in general, time to some kind of event, Dynamic Bayesian Networks allow modelling and predicting how all dynamic variables evolve in time and how these variables influence each other (in terms of conditional dependence). Therefore, a comparison with other methods is not straightforward.

However, it is legitimate to ask whether a different method would perform better or worse in terms of ability to predict, e.g., the survival time. At this purpose, we trained a LASSO ‘least absolute shrinkage and selection operator’ regression analysis [26] coupled with Cox survival model [27] and recursive feature elimination as done in [28], on the same training data used to learn the DBN model. We obtained an AU-ROC equal to 0.79 and 0.64 at months 12 and 24, respectively; at the same time points the DBN achieved an AU-ROC equal to 0.96 and 0.90. The performance dropped at following time points probably due the fact that only the first visit can be used to train the Cox-LASSO model. One of the advantages of DBNs is indeed the possibility to exploit all dynamic data information contained in the training set to learn the model.

We believe that our model provides a support for ALS prognosis in the context of a personalized medicine approach, and can be used by clinicians to stratify patients according to their most probable disease course. Note that the DBN was learnt on patients with a recent ALS diagnosis and using the clinical variables from the entire course of the disease; our next aim will be the evaluation of the performance of our model on patients that have not been recently diagnosed with ALS. Moreover, building two different models, one for long and another for short-term predictions will be considered.

As a further development, the DBN model will be tested on clinical datasets and will be made available to the scientific community as a web-based computational tool.

## Conclusions

The development of effective therapies in Amyotrophic Lateral Sclerosis is urgently required; in particular, a support of the prognosis process is crucial for decision-making and clinical interventions planning. In this study, the proposed model identified potential risk factors of ALS and simulated the dynamics of disease progression. Our method has the potential to confidently predict the outcome of patients in the four main areas of disability of ALS (communication, swallowing, gait and respiration), as well as to predict their survival.

## Additional file

Additional file 1: A PDF document with training and validation sets characteristics, quantization levels or categories of variables, and additional results. (PDF 754 kb)

## Abbreviations

ALS: Amyotrophic Lateral Sclerosis
AU-ROC: Area Under the ROC curve
CPD: Conditional Probability Distribution
CV: Cross-Validation
DAG: Direct Acyclic Graph
DBN: Dynamic Bayesian Network
HC: Hill-Climbing
MITOS: (ALS) Milano-Torino Staging system
MMHC: Maximum Minimum Hill-Climbing
PRO-ACT: Pooled Resource Open-Access ALS Clinical Trials Database
