# Supplementary material for: A Dynamic Bayesian Network model for the simulation of Amyotrophic Lateral Sclerosis progression
Source: BMC Bioinformatics. 2019 Apr 18;20(Suppl 4):118. doi: 10.1186/s12859-019-2692-x (PMC6471677; doi:10.1186/s12859-019-2692-x)
Supplement: Supplementary file 1 — A PDF document with training and validation sets characteristics, quantization levels or categories of variables, and additional results. (PDF 754 kb) [file 12859_2019_2692_MOESM1_ESM.pdf]

`

Additional file 1

DESCRIPTION	TRAINING N=3,970	VALIDATION N=987
Gender (male/female)	2,488 / 1,482	620 / 367
Age at onset (years)	56±11	55±11
Time from onset to trial start (months)	18±8	18±8
Site of onset (Bulbar/Limb/Bulbar and limb)	878 / 2,789 / 46	230 / 685 / 12
Riluzole usage (No/Yes)	919 / 2,083	237 / 515
Placebo/Treatment	1,480 / 2,196	366 / 540
Weight (Kg)	74.1±15.8	74.3±16.1
Forced Vital Capacity (%)	73.4±24.5	73.1±23.6
Chloride (mmol/L)	102.5±3.5	102.5±3.4
Aspartate amino transferase (SGOT) (U/L)	29.9±15.4	28.9±12.4
Creatine Kinase (u/L)	282.1±271.1	276.1± 248.1
White Blood Cell (10^9 cells/L)	7.0±1.9	6.9±1.9
Glucose (mmol/L)	5.6±1.6	5.6±1.7
Alkaline Phosphatase (U/L)	78.3±29.7	77.8±26.7
Calcium (mmol/L)	2.4±0.1	2.4±0.1
Hemoglobin (g/L)	144.6±12.9	144.1±12.7
Platelets (10^9 cells/L)	250.6±64.8	251.9±65.5
Sodium (mmol/L)	140.3±2.8	140.2±2.6
Blood Urea Nitrogen (mmol/L)	5.6±1.7	5.6±1.7
Potassium (mmol/L)	4.2±0.3	4.2±0.4
Bilirubin (Total) (umol/L)	9.9±6.4	10.2±6.1
Protein (g/L)	71.9±4.7	71.8±4.6
Phosphorus (mmol/L)	1.2±0.2	1.2±0.2
Alanine amino transferase (SGPT) (U/L)	34.4±23.4	33.5±23.3
Albumin (g/L)	43.2±3.6	43.1±3.5
Hematocrit (%)	43.3±3.8	43.2±3.8
Bicarbonate (mmol/L)	26.2±3.4	26.2±3.5

`

Absolute Eosinophil Count (10^9 cells/L)	0.5±1.3	0.5±1.2
Creatinine (mmol/L)	67.2±20.2	65.3±20.0
Gamma-glutamyltransferase (U/L)	35.4±38.2	35.1±42.5
Absolute Basophil Count (10^9 cells/L)	0.03±0.03	0.03±0.03

Table S1. Characteristics of subjects in the training and validation sets, before quantization

.

Variable name (type)	Description	Levels/Categories
Gender		male
		female
Age_atOnset	Age at ALS onset	age<51 (years)
		51≤age≤61
		age>61
onset_delta	Days between first symptoms and the beginning of the trial	onset<-611 (days)
		-611≤onset≤-393
		onset>-443
onset_site	Site of onset	Bulbar
		Limb
		Bulbar/limb
Riluzole	Riluzole use (also for a limited period)	no
		yes
Placebo	Placebo or medication intake	yes
		no
Movement	MITOS score	not compromised
		compromised
Swallowing	MITOS score	not compromised
		compromised
Communicating	MITOS score	not compromised
		compromised
Breathing	MITOS score	not compromised
		compromised
Weight		w<65 kg
		65≤w≤78.5 kg

`

		w>78.5 kg
fvc_percent	Forced Vital Capacity (%), volume of air that can forcibly be blown out after full inspiration	fvc<60%
		60≤fvc≤80%
		fvc>80%
Chloride	Electrolyte	chl<98 mmol/L
		98≤chl≤106 mmol/L
		chl>106 mmol/L
AST(SGOT)	Aspartate Amino Transferase	ast<7 U/L
		7≤chl≤27 U/L
		chl>27 U/L
CK	Creatine Kinase: increases may indicate a heart attack or other muscle damage	ck<38 u/L (Male); ck<96 u/L (Female)
		38-174 (M); 96-140 (F)
		ck>174 (M); ck>140 (F)
White Blood Cell (WBC)	Count of the number of white blood cells per volume of blood	wbc<4.3*10^9 /L cells
		4.3≤wbc≤10.8*10^9 /L cells
		wbc>10.8*10^9/L cells
Glucose		g<3.8 mmol/L
		3.8≤g≤6 mmol/L
		g>6 mmol/L
Alkaline Phosphatase	Also associated with bone dysfunction	alp<50 U/L
		50≤alp≤160 U/L
		alp>160 U/L
Calcium	Routine metabolic panel to assess kidney, bone, or nerve disease	c<2.2 mmol/L
		2.2≤c≤2.5 mmol/L
		c>2.5 mmol/L
Hemoglobin		h<130 g/L (M); ck<120 g/L (F)

`

	Amount of oxygen-carrying protein in the blood	130-180 (M); 120-160 (F)
		h>180 (M); h>160 (F)
Platelets		pl<150*10^9 /L cells
		150≤pl≤350*10^9 /L cells
		pl>350*10^9/L cells
Sodium		s<133 mmol/L
		133≤s≤146 mmol/L
		s>146 mmol/L
Blood Urea Nitrogen (BUN)	A product of the kidney's normal function found in urine	bun<1.2 mmol/L
		1.2≤bun≤3 mmol/L
		bun>3 mmol/L
Potassium	Electrolyte	pot<3.5 mmol/L
		3.5≤pot≤5.4 mmol/L
		pot>5.4 mmol/L
Protein	A measure of all blood protein	pr<60 g/L
		60≤pr≤84 g/L
		pr>84 g/L
Phosphorus	Associated with kidney function and nutritional status	ph<1 mmol/L
		1≤ph≤1.5 mmol/L
		ph>1.5 mmol/L
ALT(SGPT)	Alanine Amino Transferase: also associated with diseases of the biliary system	1<alt≤ 21 U/L
		alt>21 U/L
Albumin	The major protein in blood serum produced in the liver	alb<35 g/L
		35≤alb≤50 g/L
		alb>50 g/L
Hematocrit		hm<45% (M); hm<37% (F)
		45-62% (M); 37-48% (F)

`

	The percentage of red blood cells in a given volume of whole blood	hm>62% (M); hm>48% (F)
Bicarbonate	Electrolyte, associated also with acid-base (pH) imbalance	bc<18 mmol/L
		18≤bc≤23 mmol/L
		bc>23 mmol/L
Absolute Eosinophil Count	Count of eosinophils (white blood cells type) per volume of blood	0-0.5*10^9/L cells
		aec>0.5*10^9/L cells
Creatinine	Waste products filtered out of the blood by the kidneys	cr<53 mmol/L (M); cr<44 mmol/L (F)
		53-106 mmol/L (M); 44-97 mmol/L (F)
		cr>106 mmol/L (M); cr>97 mmol/L (F)
Gamma-glutamyltransferase	Enzyme found in the liver and other tissues	ggt<40 U/L
		ggt≥40 U/L
Absolute Basophil Count	Count of basophils (white blood cells type) per volume of blood	0-0.4*10^9/L cells
		abc>0.4*10^9/L cells
Survival	Subject alive at the last visit (censored) or dead during the trial	censored
		dead
Time since onset (TSO)	Months between current visit and ALS onset	tso<6 months
		6≤tso≤12 months
		12≤tso≤18 months
		18≤tso≤24 months
		24≤tso≤42 months
		tso>42 months

Table S2. Variables included in both training and validation sets, with description and quantization levels or categories.

`

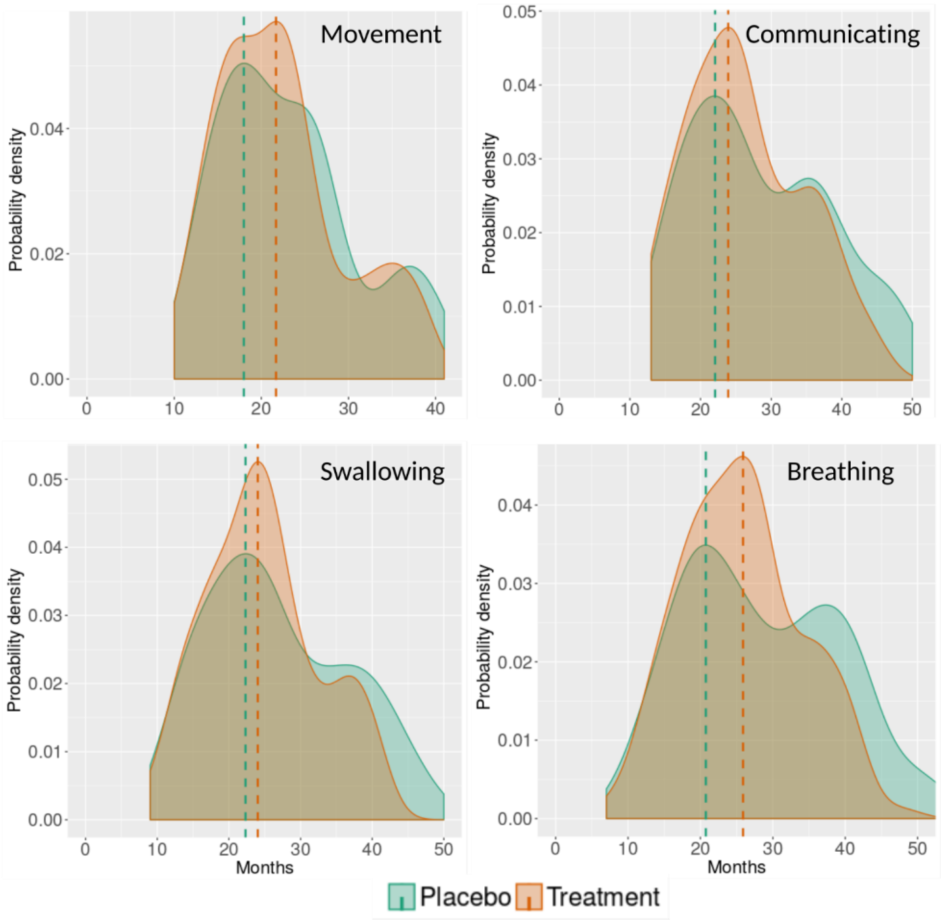

Figure S1. Density plot of the probable values (color-coded) of MITOS items along time for two simulated ALS populations with placebo (green curve) and experimental medications (orange curve) intake, respectively. 100 different temporal evolutions were generated so to have a distribution of variable values in time.
